# Silver metal nano-matrixes as high efficiency and versatile catalytic reactors for environmental remediation

**DOI:** 10.1038/srep45112

**Published:** 2017-03-23

**Authors:** Ludovic F. Dumée, Zhifeng Yi, Blaise Tardy, Andrea Merenda, Elise des Ligneris, Ray R. Dagastine, Lingxue Kong

**Affiliations:** 1 Deakin University, Geelong, Institute for Frontier Materials, Waurn Ponds, Victoria 3216, Australia; 2 University of Melbourne, Department of Biomolecular and Chemical Engineering, Parkville, Victoria 3052, Melbourne, Australia

## Abstract

Nano-porous metallic matrixes (NMMs) offer superior surface to volume ratios as well as enhanced optical, photonic, and electronic properties to bulk metallic materials. Such behaviours are correlated to the nano-scale inter-grain metal domains that favour the presence of electronic vacancies. In this work, continuous 3D NMMs were synthesized for the first time through a simple diffusion-reduction process whereby the aerogel matrix was functionalized with (3-Mercaptopropyl)trimethoxysilane. The surface energy of the silica monolith templates was tuned to improve the homogeneity of the reduction process while thiol functionalization facilitated the formation of a high density of seeding points for metal ions to reduce. The diameter of NMMs was between 2 and 1000 nm, corresponding to a silver loading between 1.23 and 41.16 at.%. A rates of catalytic degradation kinetics of these NMMS which is three orders of magnitude higher than those of the non-functionalized silver-silica structures. Furthermore, the enhancement in mechanical stability at nanoscale which was evaluated by Atomic Force Microscopy force measurements, electronic density and chemical inertness was assessed and critically correlated to their catalytic potential. This strategy opens up new avenues for design of complex architectures of either single or multi-metal alloy NMMs with enhanced surface properties for various applications.

Nano-porous metallic matrixes (NMMs) offer superior surface to volume ratios as well as enhanced optical and electronic properties to bulk metallic materials^1^,^2^, as the nano-scale metal grains favour the presence of electronic vacancies^3^.

The breakthrough properties of NMMs have been harvested to design advanced conductive pathways for nano-electronic devices, for identification of biomolecules as Surface Enhanced Raman Scattering (SERS) surfaces, or as nano-scale chemical reactors with enhanced catalytic, photocatalytic, anti-microbial or adsorptive capabilities^2^,^3^,^4^,^5^,^6^. The design of alloyed structures whereby synergistic properties such as triggered reactivity or piezo-electrical behaviours may be controlled, is of strong interest in catalysis and energy generation or storage. Nevertheless, it is more challenging since it requires a finer control of the metal grain distribution and of intercalation between metal atoms within the dominating metal matrix. Additionally, NMMs have found exotic applications as surface coatings for stimuli responsive surfaces and for visual aspect and colour control generated from variations of the Fermi level bandgaps more or less favourably bridged across the metal crystalline structures^7^,^8^. Controlling these localized energetic and mechanistic electron-pores is, however, a challenge in nano-metallurgy primarily due to the need to master the material microstructure and composition, and strongly altering crystallinity and meta-stability^3^,^9^.

Specifically, the plasmonic conduction properties, including thermal and electrical conductivities of nano-porous metal frameworks are higher than bulk metals due to the enhanced plasmonic conduction generated from the high surface to volume electronic vacancies of nanostructured metals^10^,^11^. Compared to other near 2D nano-materials (i.e. graphene or boron nitride porous sheets), key challenges for the development of NMMs lie in the control of the nature of the surface composition and particularly that of oxide, carbides and nitrides, as well as trace metal contamination present within the boundaries between metal grains, or metal organization and association including alloying effects within crystal lattices. Transition metals, such as copper (Cu), aluminium (Al) or iron (Fe), are more reactive and prone to oxidation when compared to noble metals, such as gold (Au), platinum (Pt), palladium (Pd) and silver (Ag), making them therefore much more challenging to stabilize^12^. At the nano-scale, the increased volume of grain boundaries as well as larger grain orientation distributions, induced from the materials nano-porosity, are however more likely to form amorphous metal domains with disordered atomic scale structures and thus lower cohesive properties that affect their chemical and oxidation stability^12^,^13^.

Nano-porosity across NMMs may be tuned to form single to multi-dimensional hierarchical materials with tuneable surface to volume from altering the size and shape of the nano metal grains^14^,^15^. Formation of fine and stable nano-porous shells, particles, tubes, whiskers, foils and frameworks was previously reported, primarily through bottom-up synthesis techniques involving the reductions of metal ions across a suitable template, but also through top-down routes by de-alloying of particles or thin films^16^. Non-electrochemical deposition or direct reduction of metal ions across templates is one of the most efficient strategy to form 3D NMMs materials^15^. The design of NMMs is, however, typically hindered from specific interactions between metal ions and functional groups across the surface of the template, as well as from confinement phenomena. These challenges may lead to premature pore clogging and ultimately structural deformations and damage within the template.

In this work, continuous 3D NMMs were synthesized for the first time across functionalized nano-porous silica monoliths with a novel and extremely simple diffusion-reduction process. Ag ions firstly diffused across the silica framework with reducing agent (ethylene glycol) in order to obtain uniform Ag ion distribution through silica framework. Subsequently the whole system was heated to 160 °C to induce the reduction of Ag ions to Ag nanoparticles which in turn form Ag network in silica aerogel. The main novelty of this work is to provide a new route to the formation of continuously porous NMMs with great control on the reduction and diffusion rates of the silver ions source. Nano-porous silica materials, synthesized through sol-gel processes, whereby silicon alkoxides are hydrolysed and condensed, offer high surface to volume ratio and extremely porous structures^17^. The alkoxide chains may be easily functionalized to form anchoring points for doping or specific encapsulation. Furthermore, while a large array of silica networks may be generated depending on the relative rates of reactions, the type of alkoxide precursor and the operating process conditions will affect both reactivity and wettability to salts. Silica materials are naturally hydrophilic and were previously shown to exhibit high metal ions uptake^18^ due to their negative surface potential that can favourably interact with, and reduce, metal ions^19^. The homogeneity of the reduction procedure was found to be largely dependent on the surface energy of the silica framework and on the presence of favourable seeding points for the reduction of metal ions. The application of these NMMs as catalytic reactors for the remediation of toxic bio-chemicals is demonstrated with extremely high kinetics. The enhancement in mechanical stability at the nanoscale, evaluated by Atomic Force Microscopy (AFM) nano-indentation, the electronic density, and chemical inertness was assessed and critically correlated to their catalytic potential.

## Results and Discussions

The growth mechanism of the NMMs follows a direct pathway first involving the homogeneous nucleation of Ag atoms^20^, prior to progressive growth of silver nanoparticles that ultimately bridges into a continuous network. 15-Mar-2017 12:08 S1 shows the schematic of growth mechanism of NMMs. Ag ions were firstly diffused across the silica gel in ethylene glycol that performed as both solvent and reducing agent^20^. The modification with MPTMS brought thiol groups on the surface of silica framework, offering the arching points to preferentially adsorb Ag ions and Ag nanoparticle nuclei^21^. The growth of Ag nanoparticles then started from the surface bonding with thiol groups and then extended to the centre of channels formed by silica. With continuous growth and diffusion from the bulk solution, Ag network was formed.

**Figure 1:**
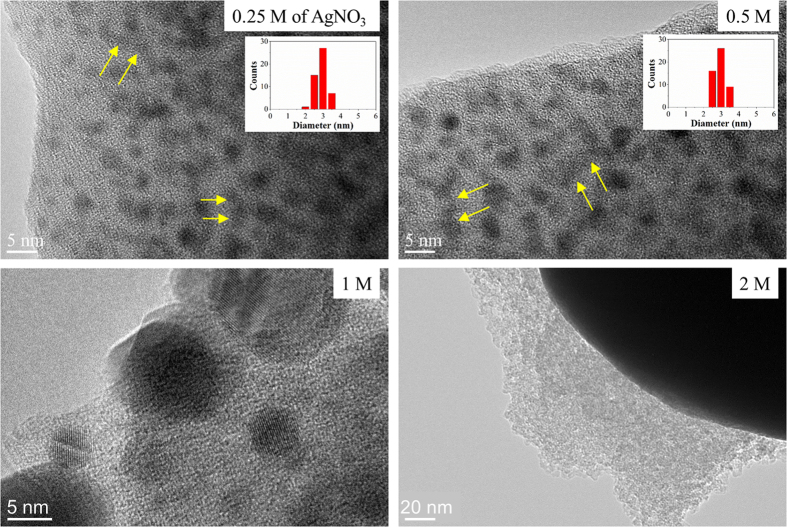
High magnification TEMs of the hybrid MPTMS – silica – NMMs aerogels with different concentrations of AgNO_3_ at 0.25, 0.5, 1 and 2 M. The coalescence of the network is highlighted with yellow arrows on the images. The insets are the corresponding size distribution of silver nanoparticles grown in silica network.

As seen across the series of TEM images shown in Fig. 1 and Figure S2 (Supporting materials), an increase in concentration of silver ions within the matrix led to the formation of continuous metal bridges within the pores of the bare silica material. The NMMs were also found to be stabilized with specific functionalization of the silica surface with MPTMS. The surface grafting of MPTMS, which was previously shown to simultaneously reduce the pore size and volume across the silica aerogel and alter the surface charge and reactivity of silica surfaces^5^,^22^, was found to form more homogeneous NMMs with a higher density of nano-sized particulates (Figs 1 and S2). At MTPMS concentrations below 1 M, this was primarily attributed to the increased concentration of metal nano-particle seeds occurring after MPTMS grafting. The size distribution of the particles at the same AgNO_3_ salt concentration was found to be much narrower, on the order of 3 nm (Insets in Fig. 1) compared to hundred nanometres across the bare silica materials. Percolation of the silver network across the large scale aerogel may however have been partially hindered upon progressive reduction of the metal ions. Specifically, although the aerogel was initially impregnated with metal ions, the diffusion of the reducing agent may have led to a skin-core structure thus forming a denser network on the edge of the monoliths. This phenomenon may have been enhanced for the MPTMS samples since smaller channels were made available for liquid and ion diffusion due to the thickness of the grafted layer as previously reported for other type of silane grafting across silica matrixes^23^. However, above 1 M of AgNO_3_, the particle size was found to increase dramatically likely due to the quicker nucleation and high contacting chance between already formed particles and ions at higher Ag + concentrations^24^. Optical images in bright and dark fields (Figure S3) confirmed the visual aspect of the silica monoliths upon silver reduction, with a progressive darkening of the surface and a spatially homogeneous enhancement of the optical reflectance of the material with increasing silver content. The saturation of silica matrix attributed to the premature precipitation and reduction of metal ions however appeared to be reached for both series of samples at 1 M of AgNO_3_ salt concentration. Although, densification of the network was found to still occur up to 2 M, larger shells were found to form on the outer layer of the silica monoliths (Figure S4).

**Figure 2:**
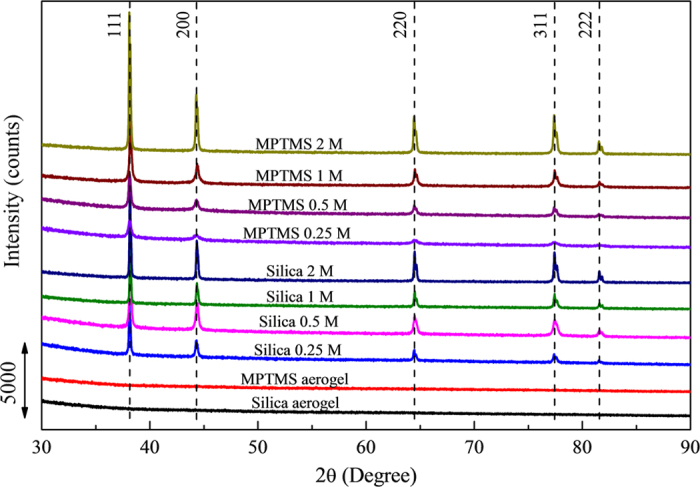
XRD of the silica, MPTMS functionalized and series of silver aerogels. As expected no peaks at 37, 44, 64, 77 and 82 degrees could be detected across the native silica and reference MPTMS aerogel samples. All graphs are plotted at the same scale.

**Figure 3:**
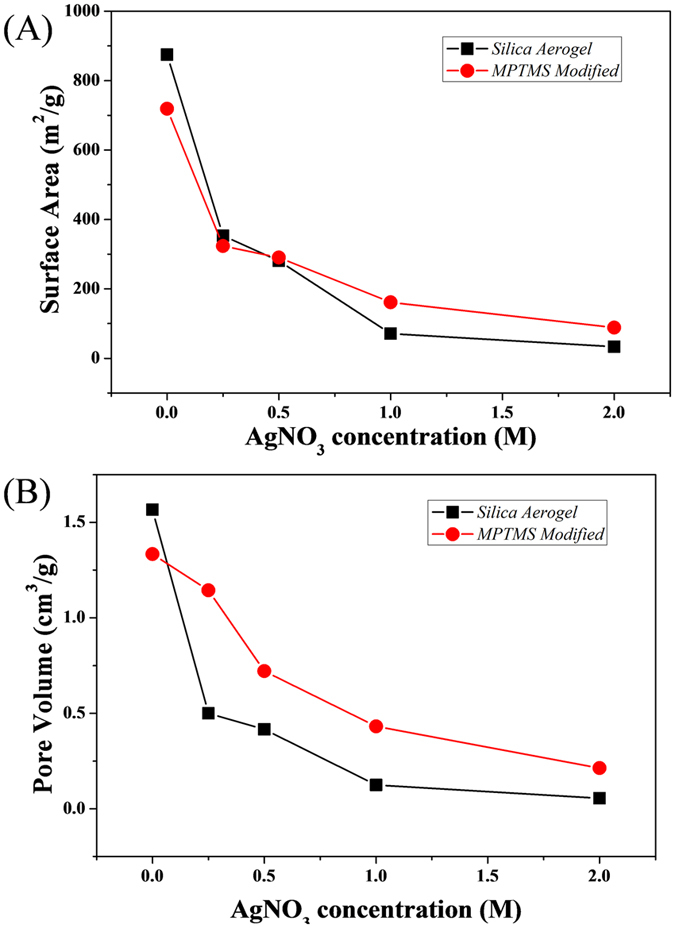
(**A**) BET specific surface area for both hybrid silica and MPTMS aerogels samples and (**B**) corresponding pore volume measurements.

The crystallinity of the NMMs, with consistent diffraction peaks at 38, 44, 64, 77 and 82°, corresponding to the Ag lattice (111), (200), (220), (311) and (222) respectively, were clearly visible on the XRD patterns shown in Fig. 2. The calculated crystallite size from XRD results is shown in Table S1 according to Scherrer equation^25^. The crystallite size increases at the main diffraction peaks (111), (200), (220) and (311) with the increasing AgNO_3_ concentration, which indicates similar trend to the increasing particle size obtained from TEM. The reason why these two techniques gave significantly different size range might be that Ag nanoparticles tend to be coalesced with each other during the growth and the crystal structure of individual Ag nanoparticles is not monocrystalline^26^, as the visible network formation observed from TEM images. Also, there is peak splitting at 64, 77 and 82° from the XRD results, which could be attributed to the neighbouring Ag crystallites forming different lattice orientation, in turn leading to a slight splitting of the diffraction peaks. Furthermore, the silver content was also extremely well correlated to the intensity of the main Ag peaks visible on the XRD patterns which had increased by 1.5 and 5 times for the bare silica and MPTMS functionalized samples respectively, between the 0.25 and 2 M samples (Fig. 2). This increase in Ag content was confirmed by the progressive reduction of both the specific surface area and free pore volume across the materials upon silver reduction as seen in Fig. 3. The specific surface area of the samples was reduced by 96% from 874 to 33 m^2^/g between the bare silica reference frameworks and the 2 M samples, while it was reduced by 87% from 719 to 88 m^2^/g between the MPTMS reference and the 2 M functionalized samples. Similarly, the pore volume reduced with Ag loading, demonstrating the filling of the silica aerogel pores. The average pore volume decreased from 1.5 to 0.05 cm^3^/g for the silica aerogel series and from 1.3 to 0.2 cm^3^/g for the MPTMS series. The extremely low pore volume remaining after Ag loading and the plateau of the specific surface area and pore volumes occurring between 1 and 2 M suggests a high level of ramification of the network. This trend could be related to the aforementioned densification and percolation threshold limit of the system, whereby nano-cages of silver/silica may have been formed upon premature reduction of the metal ions on the outer rim of the samples. The formation of these cages however appears to be limited since the overall surface area of the samples remained significantly high, which gives high metal loading after loading with silver ions. The former observations are supported by the TEMs analysis where the 3D network was found to be continuous beyond 1 M. Interestingly, the MPTMS samples were found to undergo a drastically lower reduction of their pore volume suggesting a more uniform and less significant clogging of the channels within the frameworks than for the bare silica. The MPTMS functionalization therefore appears to facilitate the wettability of the silica matrix, leading to a better diffusion of the Ag^+^ ions across the material. The MPTMS functionalized aerogels also provided anchoring points for the coordination and reduction of the metal ions, thus leading to more stable and yet still permeable frameworks.

**Figure 4:**
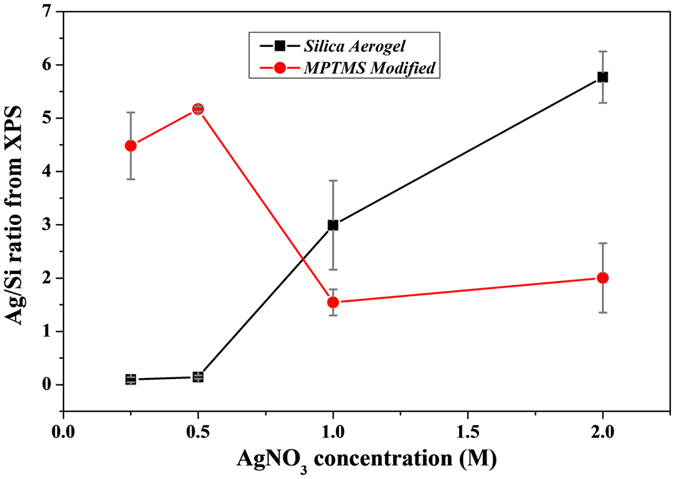
Ag/Si atomic ratios obtained from XPS data analysis.

The Ag concentration within the material was also measured with XPS by monitoring the Ag/Si ratio to evaluate the overall uptake within the silica monoliths. The monoliths were crushed into a fine powder of sub-micron size for this purpose to provide an idea of the homogeneity of the Ag distribution. As seen in Fig. 4, the overall Ag content for both bare silica and MPTM grafted samples were found to be different confirming the role of the MPTMS grafting (full spectra is provided in Figure S5). The Ag loading increased semi-linearly with the Ag^+^ ion concentration while the maximum concentration for the MPTMS samples was found around 0.5 M prior to plateauing at a value nearly 70% lower than that obtained at 0.5 M. This trend could be attributed to the formation of larger particles at higher Ag^+^ concentrations (1 and 2 M respectively), which in turn lead to the faster depletion and to a reduction of the Ag density in the centre of the monolith. A gradient of silver density was therefore visible across the cross section of the monolith at these high concentrations. However, at lower concentrations, dense and homogeneous silver networks were formed (0.25 and 0.5 M), as visible across TEM images in Figure S2. The higher loading for the 0.5 M Ag^+^ samples also suggests that Ag^+^ diffusion may have been hindered during the reduction process, due to the better filling of the silica nano-pores and the better initial wettability and reduction capability of the matrixes, provided by the MPTMS grafting. The diffusion rate across the networks was therefore likely hindered due to the inaccessibility of some pores upon clogging from the reduced metal matrix. The rate of diffusion was therefore strongly reduced for these sizes. An opportunity to improve the homogeneity across the materials may arise from the design of smaller monoliths. Although not investigated in this work, the formation of capillary-type monoliths for instance, may support the diffusion-reduction kinetics and thus facilitate the formation of longer range order matrixes.

**Figure 5:**
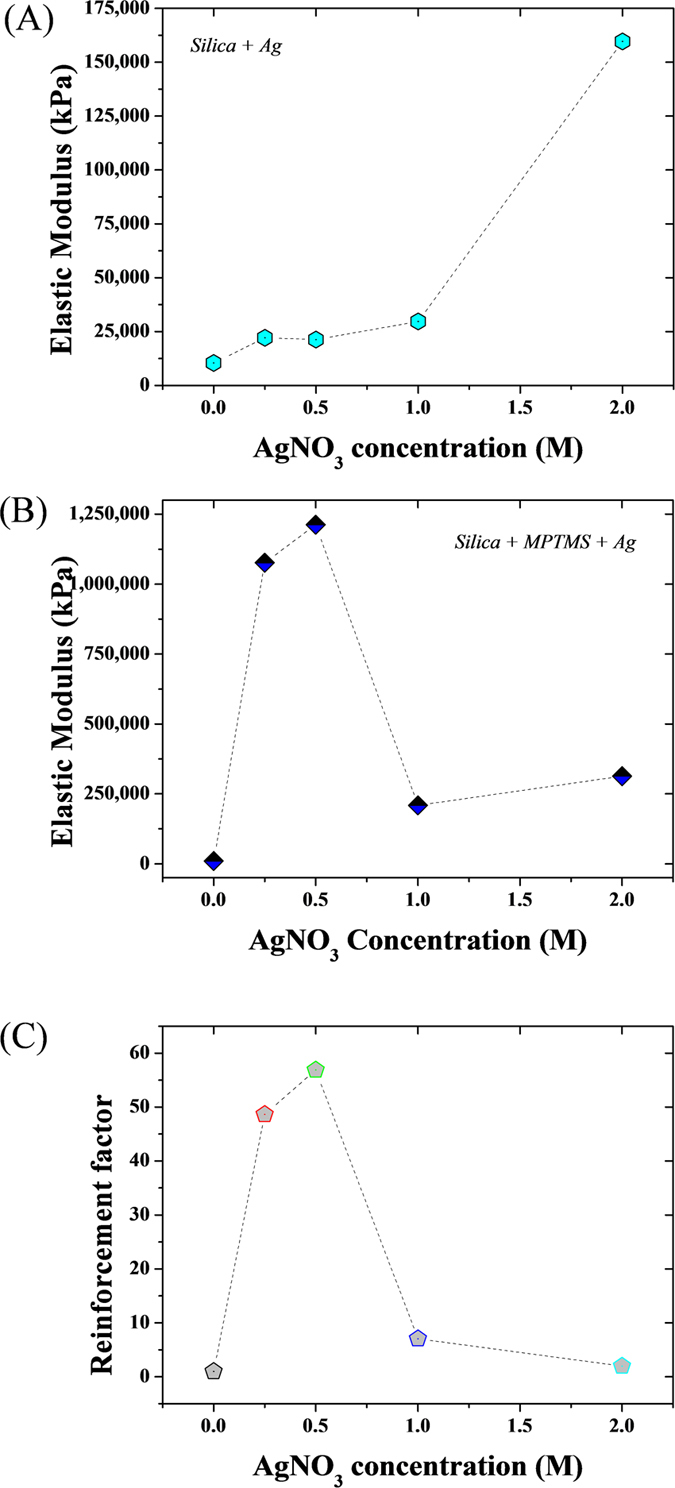
AFM force measurement data analysis for the hybrid (**A**) silica and (**B**) MPTMS aerogel series. (**C**) Absolute enhancement factors between the MPTMS and the native silica samples highlighting the benefits of the MPTMS functionalization.

The enhancement in mechanical properties of the silica-MSNs frameworks was revealed by AFM-based nano-indentation (Fig. 5). Values obtained using a conical indenter are reported in Figure S5B. Although variations in synthetic conditions generally lead to different densities and thus mechanical properties of the aerogels, the value obtained for the elastic modulus of the bare aerogel used herein is 10.4 MPa which is close to the values reported in the literature (Figure S5B)^27^,^28^. The elastic modulus of the hybrid MPTMS grafted silica frameworks were up to 55 times higher than that of the bare silica ones, at similar Ag content (representative curves in Figure S5). This result is again suggesting a more homogeneous and continuous formation of the NMMs within the MPTMS grafted silica frameworks. The presence of the MTPMS appeared to render the aerogel harder, thus reinforcing the structure and reducing the chance for mechanical rupture upon metal matrix formation. The mechanical stability of aerogels is typically a critical factor limiting their application and development, and the current strategy provides a route to both facilitate wetting and improve the material stability. The improved surface deposition and likely reduction mechanisms may have stimulated the formation of a more intimate silica/metal interface thus leading to a much more elastic material. The interface between the silica matrixes and the Ag^+^ ions is critical to ensure the formation of a flexible material in order to benefit from the NMMs 3D architecture. For the NMMs prepared with MPTMS, while the range of elastic moduli measured varied significantly around the average, the values were all of the same order for each respective metal increment. This is in contrast with the NMMs prepared without MPTMS where some areas were measured to have mechanical properties that are very similar to bare aerogels (Figure S6). This suggests an incomplete network, where percolation was not reached. The proposed method, whereby MPTMS was used as a molecular wetting agent within the aerogel structure provides a new route to increase further that percolation threshold. Furthermore, the large reduction in elastic moduli observed for 1 M and 2 M compared to 0.5 M in the presence of MPTMS correlates well with the silver content reported in Fig. 4. This trend further confirms the reduced integrity of the network when the concentration of silver is high enough to form a diffusion barrier at the edges of the material.

**Figure 6:**
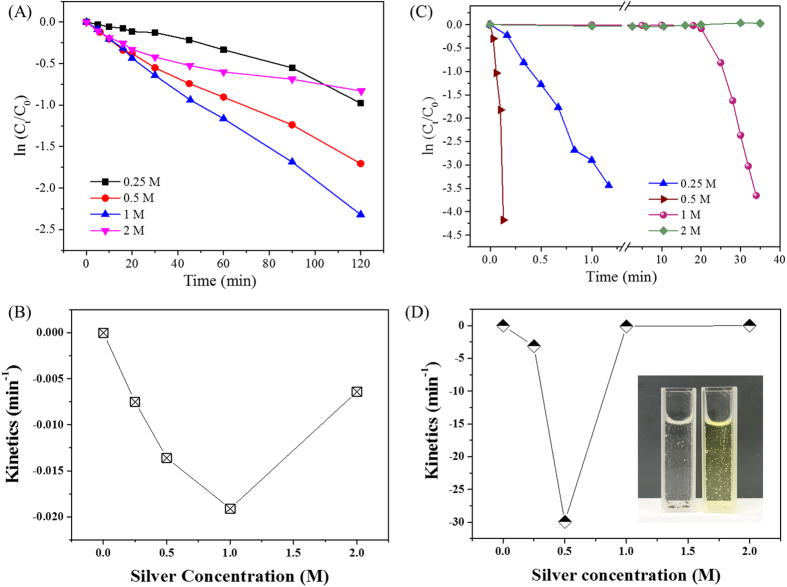
Catalysis experiments with 4-NP as the probe molecule. (**A**) Bare Ag loaded silica aerogel concentration and (**B**) kinetics profiles; (**C**) MPTMS functionalized frameworks concentration and (**D**) kinetics profiles. The inset in D is a photo of 4-NP solution in a cuvette after and before reduction. The absence of colour (left cuvette) indicates the complete reduction of 4-NP with NMM catalysts.

The Ag NMMs were also found to act as a stable and highly efficient catalyst to degrade 4-nitrophenol, a model molecule for catalysis test^4^. As shown across Fig. 6, the highest degradation kinetics were achieved at Ag^+^ equivalent concentrations between 0.5 and 1 M for the bare silica aerogels and between the 0.25 and 0.5 M for the MPTMS samples (Figure S7). Particularly remarkable, the kinetics of the 0.5 M MPTMS samples were found to go as high as 30 min^−1^ (a video indicating the vigorous reaction has been shown in Video Supporting Information), which was over 1000 times higher than the bare silver decorated silica gels and over 10 times higher than the second best sample, 0.25 M, in that series. The presence of the MPTMS likely increased the silver NPs density, thereby enhancing the catalytic behaviour (Figs 1 and S2), which is attributed to the higher reactivity of the nano-scale Ag metal grown across the MPTMS grafted matrix. As seen in Fig. 6, the catalytic potential of the materials was found to decrease with increasing Ag content (Fig. 4) confirming the densification of the network and the formation of larger particles, as seen across the TEM series for the bare silica aerogels compared to the MPTMS grafted ones.

**Figure 7:**
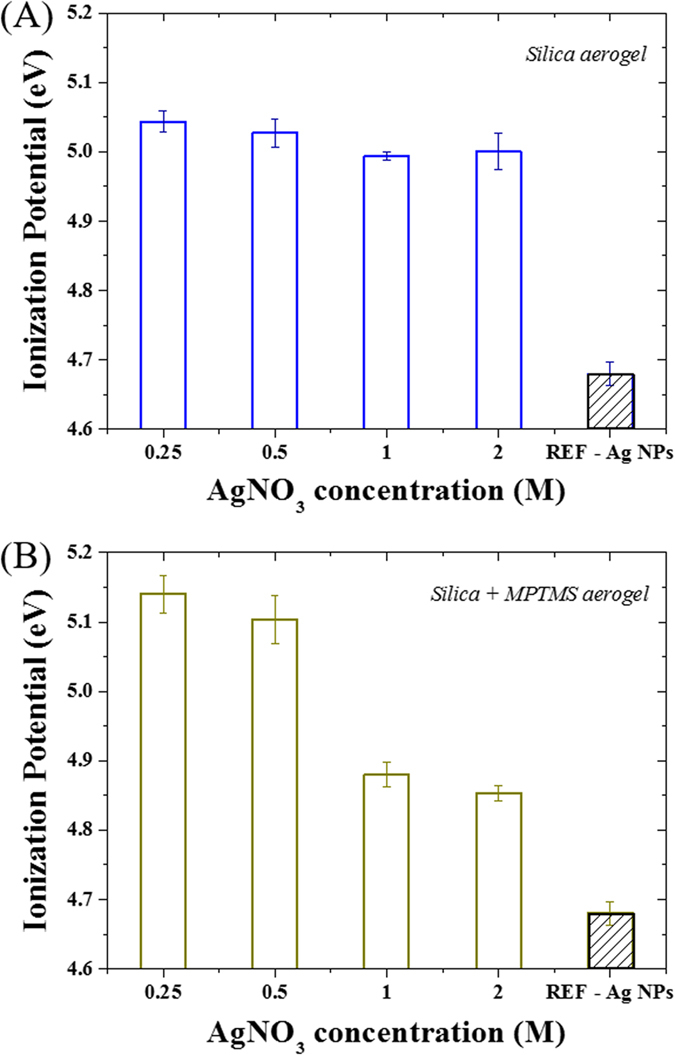
PESA analysis and ionization potential for both the (**A**) silica and (**B**) MPTMS hybrid aerogels. No ionization potential could be detected for silica. Reference metal Ag NPs are provided as a reference to assess the benchmark value of the pure silver material.

The ionization potential (IP) of the materials was also found to be largely affected by the surface functionalization (Fig. 7). The IP was found to slightly decrease for the bare silica aerogel series from 5.05 to 5 eV, while a much sharper decrease was achieved for the MPTMS series from 5.13 to 4.85 eV. These results, when compared to the reference, 20 nm diameter AgNPs, exhibiting an IP of 4.65 eV, reinforce the view that smaller grain size were achieved upon MPTMS grafting. This aspect is again supporting the fact that a balance of the silver concentration must be reached to ensure the formation of a continuous silver matrix while preventing clogging and inhomogeneous grain size/particle size distribution.

Therefore, as described from Figs 1 to 6, while the pore sizes and surface area remained high upon reduction of silver on the silica matrixes, the preferential deposition of silver at the edges of the matrixes at high concentrations resulted in a reduced homogeneity of the silver network. This aspect of the reduction process translated into the fact that the time-dependent accessibility of these pores is not homogeneous across the samples. This result highlights the constraints pertaining to the formation of NMMs from a support (herein functionalized silica aerogel). With this technique, the pore needs to remain accessible rapidly by diffusion for a high catalytic rate. The presence of a high amount of silver is required for better kinetics but the overabundance of silver, specifically at the outer edges of the materials may limit the diffusion of the reactants. Thus NMMs formed by diffusion based reduction as described herein can results in extremely high kinetics improvements but only through optimization of the network’s access and wettability.

## Conclusions

The formation of continuous Ag NMMs was demonstrated by specific reduction of Ag ions across a silica aerogel. The ramifications of the metal arms across the materials were found to extend within the frameworks and form semi-dense networks with very likely skin-core structures due to radial densification upon reaching percolation threshold. The specific surface area and diffusion-limited access of the materials could be finely tuned to generate versatile catalytic materials and the NMMs potential for degrading 4-NP was demonstrated with up to 30 min^−1^ of catalytic degradation rate, one of the highest reported to date for Fenton-like catalytic systems. The versatility of this approach also opens up new avenues for the design of large scale nano-porous metal alloy materials with enhanced plasmonic properties with potential application in optics, surface enhanced Raman scattering, separation and catalysis, where performance may be tuned from band-gap energy alterations or specific control of surface atomic vacancies.

## Methods

### Materials

Tetraethylorthosilicate (TEOS, 98%), ammonium fluoride, ammonium hydroxide, ethanol, silver nitrate, ethylene glycol, (3-Mercaptopropyl)trimethoxysilane (MPTMS), 4-nitrophenol and sodium borohydride (NaBH_4_) were purchased from Sigma Aldrich. All chemicals were of analytical grade and used without further purification. Silver nano-particles (Ag NPs) were used as a control for ionization potential measurements and catalysis tests. The particles were purchased from NanoAmor (Houston, TX, USA) and exhibited an average diameter of 20 nm for a purity > 99.9 at%.

### Synthesis of Silica Aerogel and MPTMS Modified Silica Aerogel

The silica gel frameworks were fabricated through a one-pot sol-gel procedure as previously reported^17^. The stock catalyst solution was made from 1.85 g of ammonium fluoride, 22.78 mL of ammonium hydroxide solution and 100 mL of deionised water. In a typical synthesis procedure, 0.7 mL of TEOS was mixed with 1.54 mL of ethanol as the first solution, while 0.99 mL of deionised water, 0.05 mL of catalyst solution and 1.54 mL of ethanol were mixed as the second solution. The second solution was then added into the first solution progressively and stirred until forming a uniform mixed phase. The mixture was poured into 4 syringe moulds of 1 mL each. The gels were formed within 30 min and were aged at room temperature for 24 h before being transferred into absolute ethanol for purification. The gels in ethanol were heat treated in an oven at 80 °C for 24 h. Samples were rinsed in ethanol up to 3 times, collected and stored in ethanol. To obtain aerogels all samples were dried with a critical point dryer (Leica EM CPD300) using liquid carbon dioxide. The MPTMS modified silica gel was fabricated through a co-condensation process with MPTMS and TEOS. To the first solution, 0.1 mL of MPTMS was added and mixed with TEOS and ethanol. The rest of the procedure was the same as that used to synthesize silica gel framework. The final samples were stored in ethanol until further use.

### Growth of Silver in Silica Gel and MPTMS Modified Silica Gel

Silver (Ag) ions were reduced across the silica frameworks to form the NMMs. Ethylene glycol was used as reducing agent. In details, silica or MPTMS modified gel was exchanged with fresh ethylene glycol for 6 times through 48 h to ensure the ethanol has been removed from the silica framework. Then, the gels in ethylene glycol were transferred to a silver nitrate ethylene glycol solution with a certain concentration (0.25, 0.5, 1 and 2 M). After 24 h of immersion, the samples were heat treated at 160 °C in an oven for 3 h to reduce the silver ions within the silica framework. The transparent gels turn into white or dark colour depending on the concentration of silver nitrate and surface functionalization. All the final gels were exchanged with absolute ethanol for 6 times over 48 h to remove ethylene glycol and unreacted silver ions, before dried with a critical point dryer to obtain aerogels. The shape of the aerogel was well maintained after the reduction of silver ions.

### Materials Characterization

Transmission Electron Micrographs (TEM) were acquired on a JEM-2100 transmission electron microscope (JEOL, Japan) operating at an accelerating voltage of 200 kV. The aerogel samples were cut into small pieces and immersed in absolute ethanol. The suspended sheets in solution were dropped on to carbon film coated holy copper grids and dried for imaging. Nitrogen adsorption–desorption isotherms were acquired on a Micromeritics Tristar 3000 analyzer (Particle & Surface Science, UK) at 77 K under continuous adsorption conditions. Prior to measurement, all samples were degassed at 110 °C for 4 h under nitrogen gas flow. Pore volume and specific surface area were calculated using the Brunauer-Emmett-Teller (BET) and Barrett–Joyner–Halenda (BJH) methods. Optical microscopic images were obtained from a DP71 microscope system at 20x and 100x magnification (Olympus, USA). All aerogel samples were split into small pieces and wetted with absolute ethanol for observation. X-ray powder diffraction (XRD) patterns were obtained in the range of 2θ from 5 to 90° using a Phillips PW-1729 diffractometer (40 kV, 30 mA) (Amsterdam, Netherlands) with CuKα radiation (λ = 0.154 nm). Atomic Force Microscopy (AFM) analysis was performed on an Asylum MFP-3D. Indentation experiments were performed with a spherical probe of radius of 5.35 μm and with a tetrahedral probe with a half cone angle of ~25 degree. While conical indentors gave the best results because of a larger, more homogeneous indentation, spherical indentors lead to a higher error (Figure S6A). This is most likely the result of a high surface area on initial indentation where roughness affects the measurement significantly as previously reported^27^. The indentation was performed over a deflection of at least 200 nm at a speed of 2 μm per second. Aerogels were broken into pieces of ca. 100 μm for analysis. Flat faces were subjected to indentation. When necessary, the aerogels were immobilised on double sided tape, this did not to affect the moduli measured but improved consistency between the measurements. Without tape, occasionally, aerogels chunk would tip over slightly during indentation. Aerogels were placed on a glass slide and then indented after calibration of the cantilevers. Spherical probe experiments yielded data with large discrepancies, probably due to the roughness of the sample. Furthermore, the use of a spherical probe resulted in the presence of large, long range electrostatic effects. Generally increasing with an increase of metal fillers inside of the silica aerogels. Overall, the trends were conserved going between spherical and conical shapes but conical shape resulted in higher elastic moduli and a smaller variance of the data. The issues with the spherical probe are attributed to a high roughness, static and a low pressure due to a higher surface area resulting in smaller indentations. Data are reported using the Oliver-Pharr analysis^29^ with a semi angle of 25 degree, an intercept factor sigma of 0.75 and a geometry correction factor beta of 1.034^30^. The data presented are the result of at least 10 indentations in 10 different areas of the materials analyzed. The XPS analysis was carried out at the Australian Microscopy and Microanalysis Research Facility (AMMRF), RMIT University, using a K-Alpha X-ray photoelectron spectrometer (Thermo Fisher Scientific). An Al Kα (1486.6 eV) X-ray source was used as the excitation source, and the anode was maintained at 250 W, 10 kV, and 27 mA at a chamber pressure of 1 × 10^−9^ ± 0.1 Pa with an oval beam spot size of 400 μm × 400 μm. The spectra were acquired on 10 scans and a pass energy of 100 eV on 3 different spots for each sample. The data was evaluated by using CasaXPS software with the introduction of Shirley backgrounds and Relative Sensitivity Factors (RSF) for each element in accordance with the CasaXPS library. The ionization potential was measured using Photo-Electron Spectroscopy in Air (PESA – Model AC-2). In this technique UV light is used to generate photo-electrons which ionize atmospheric oxygen molecules that are accelerated towards an open counter detector. By plotting the yield (n = 0.33 or 0.5 for semiconductor or metal materials, respectively) versus energy, the ionization energy can be determined. A UV intensity of 50 nW, UV energy between 4.2–6.2 eV with 0.0031 nW step, and n = 0.5, was used. For each sample at least 3 measurements were performed on different areas and the average taken.

### Catalytic Performance

The catalytic reduction of 4-nitrophenol (4-NP) by NaBH_4_ was chosen as a model reaction to investigate the catalytic capacity of the aerogel samples. Following a previous procedure^4^, in a typical experiment, 30 mL deionised water and 750 μL of 4-nitrophenol (3 mM) were mixed with 1 mL of freshly prepared NaBH_4_ (300 mM). A solution volume of 3 mL was transferred into a quartz cuvette. Two mg (+/−0.1 mg) of powder samples were immediately added into the quartz cuvette. The *in situ* UV-Vis absorption profiles were recorded with an Ocean optics USB2000 Miniature Fiber Optic Spectrometer with PX-2 Pulsed Xenon Light Source in the range of 250–550 nm to monitor the catalytic reaction and kinetics of degradation of the 4-NP by recording over time the intensity of the peak at 400 nm.

## Additional Information

**How to cite this article:** Dumée, L. F. *et al*. Silver metal nano-matrixes as high efficiency and versatile catalytic reactors for environmental remediation. *Sci. Rep.* **7**, 45112; doi: 10.1038/srep45112 (2017).

**Publisher's note:** Springer Nature remains neutral with regard to jurisdictional claims in published maps and institutional affiliations.

## Supplementary Material

Supplementary Information

Supplementary Video
