# Supplementary material for: Silver metal nano-matrixes as high efficiency and versatile catalytic reactors for environmental remediation
Source: Sci Rep. 2017 Mar 23;7:45112. doi: 10.1038/srep45112 (PMC5362805; doi:10.1038/srep45112)
Supplement: Supplementary Information [file srep45112-s1.pdf]

Supplementary Information

Title

Silver metal nano-matrixes as high efficiency and versatile catalytic reactors for environmental remediation

Authors

Ludovic F. Dumée[1*$], Zhifeng Yi[1$], Blaise Tardy[2], Andrea Merenda[1], Elise des Ligneris[1], Ray R. Dagastine[2], Lingxue Kong[1]

Affiliations

1 Deakin University, Institute for Frontier Materials, Waurn Ponds, Victoria 3216, Australia

2 University of Melbourne, Melbourne, Department of Biomolecular and Chemical Engineering, Parkville, Victoria 3052, Australia

[*] ludovic.dumee@deakin.edu.au; +61410131312

[$] These authors contributed equally to the manuscript

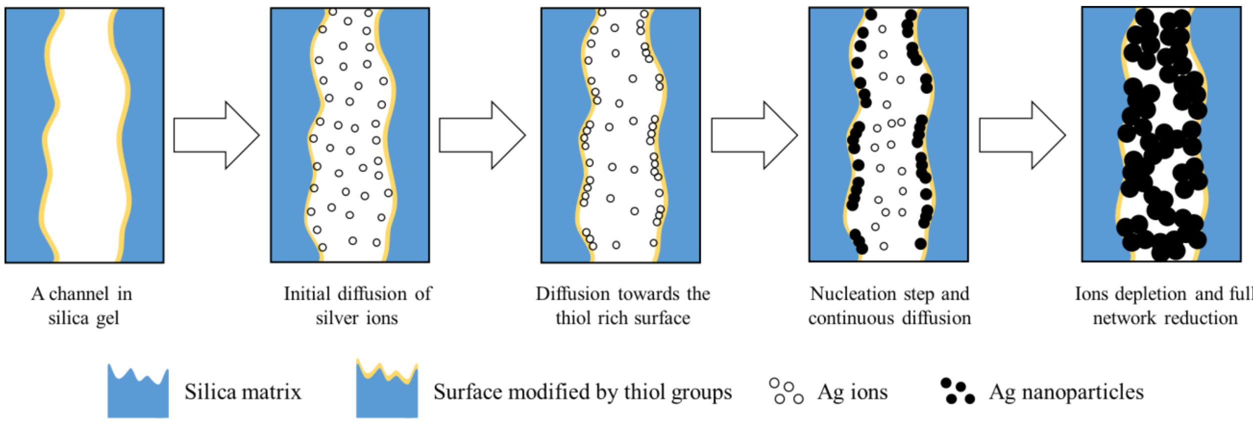

Figure S1 Schematic of the growth mechanism of Ag nanoparticles in MPTMS modified silica aerogel.

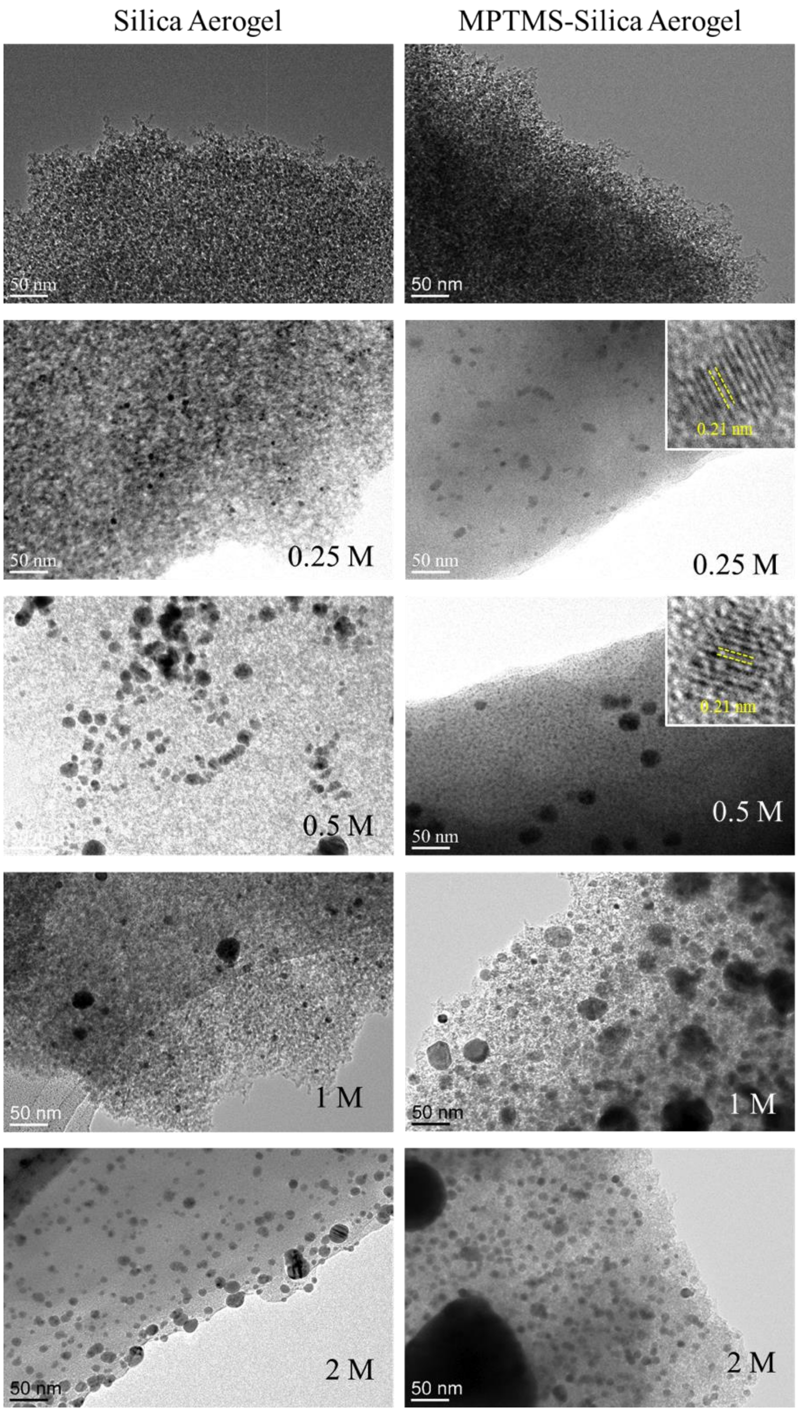

Figure S2 TEM images of the (A) bare silica and (B) the reference MPTMS functionalized aerogels. The formation of high density particles and their progressive coalescence is highlighted as a function of the Ag concentration. Representative crystallinity of the as-generated Ag lattice (distance 0.21 nm) is shown across the inserts in the samples grown with 0.25 M and 0.5 M $AgNO_3$ in two MPTMS samples.

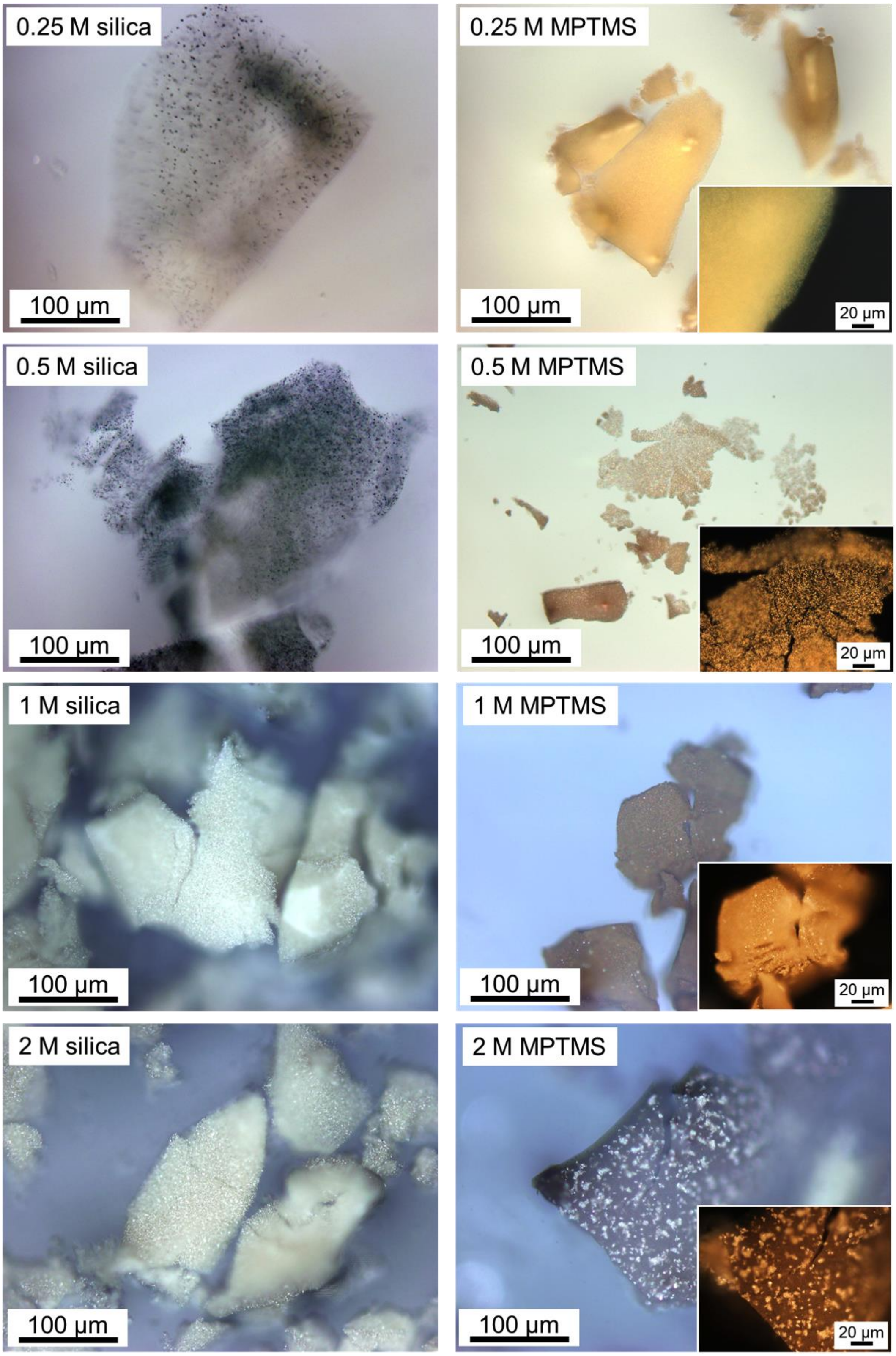

Figure S3 Optical microscope images of the series of silica and MPTMS functionalized hybrid aerogels in bright field and dark filed (insets).

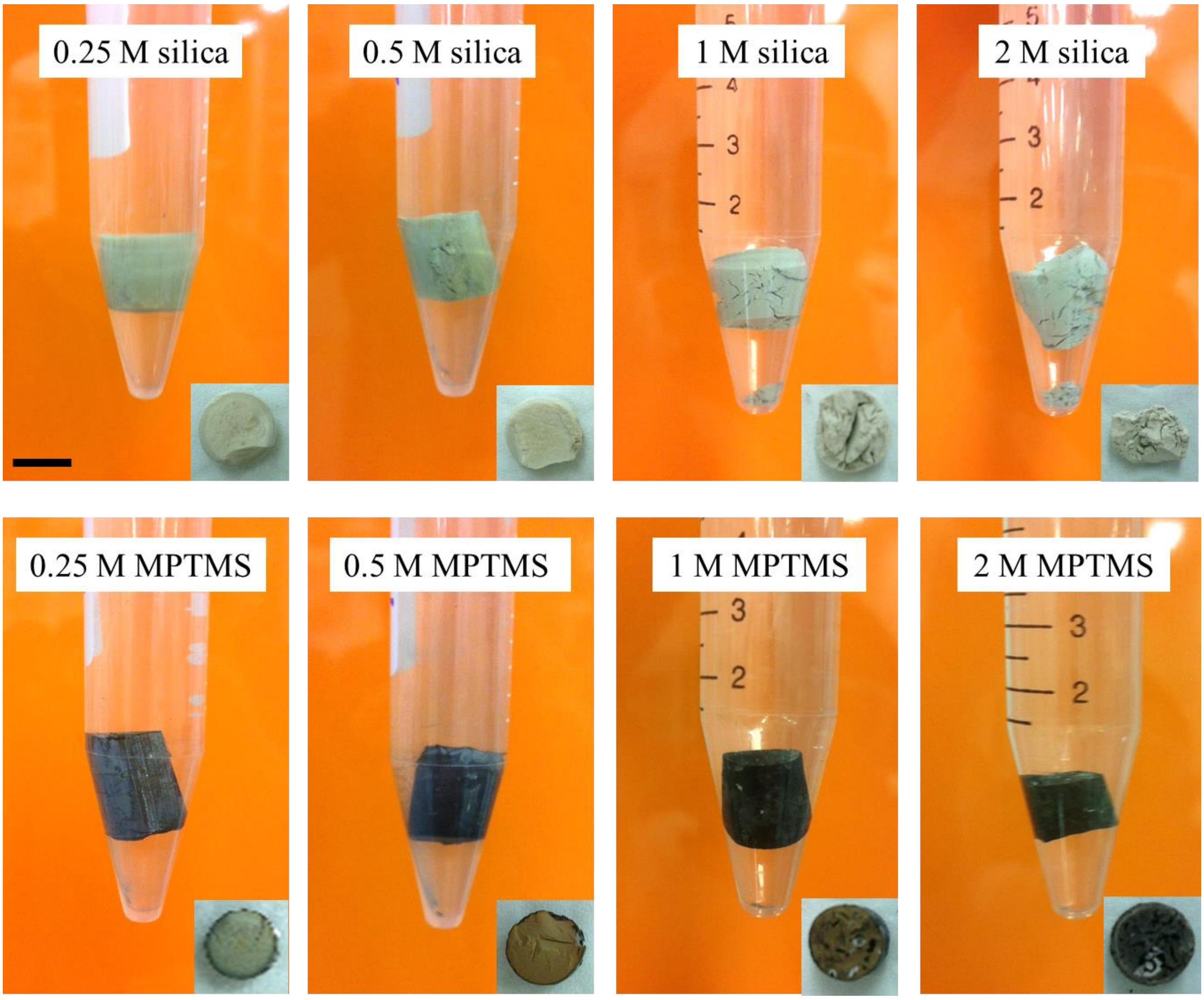

Figure S4 Visual aspect of the silica – NMMs monoliths for various concentrations of silver nitrate in silica hydrogel (top) and MPTMS modified silica hydrogel (bottom). The insets are the cross-section of corresponding aerogels after drying in a critical point dryer. The scale bar is 1 cm. The samples grown at higher concentration of silver nitrate (1 M and 2 M) tend to be collapsed in both bare silica and MPTMS modified aerogels.

(A)

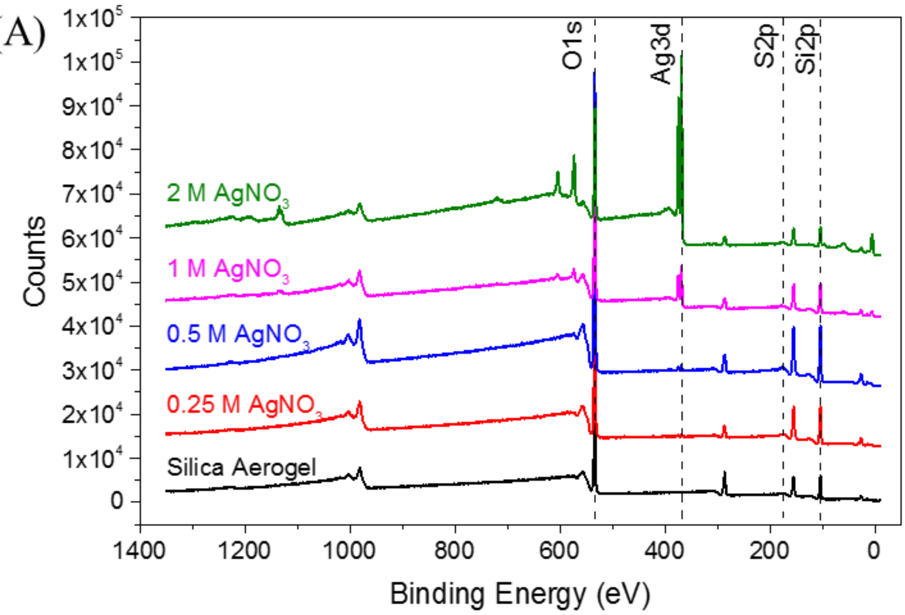

(B)

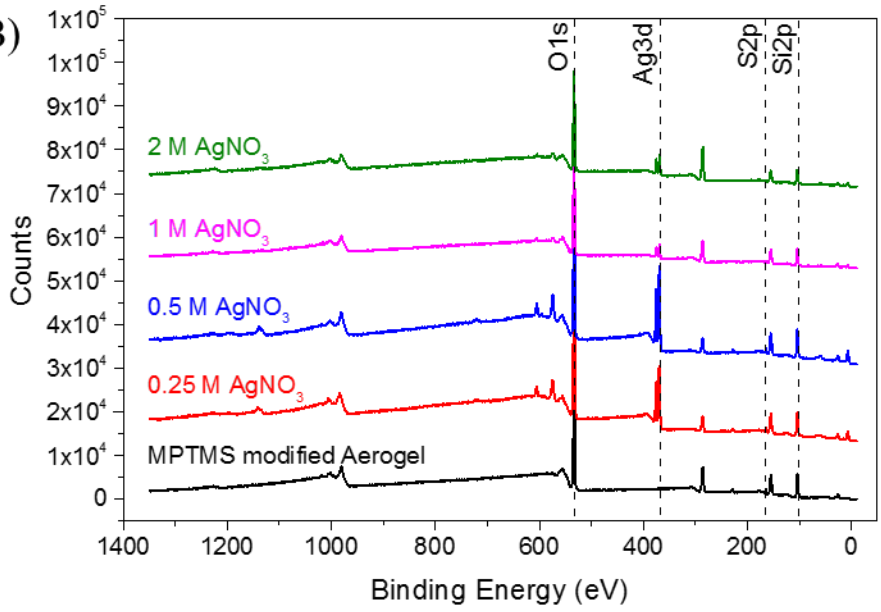

Figure S5 XPS survey scans for (A) silica and (B) MPTMS functionalized hybrid aerogels.

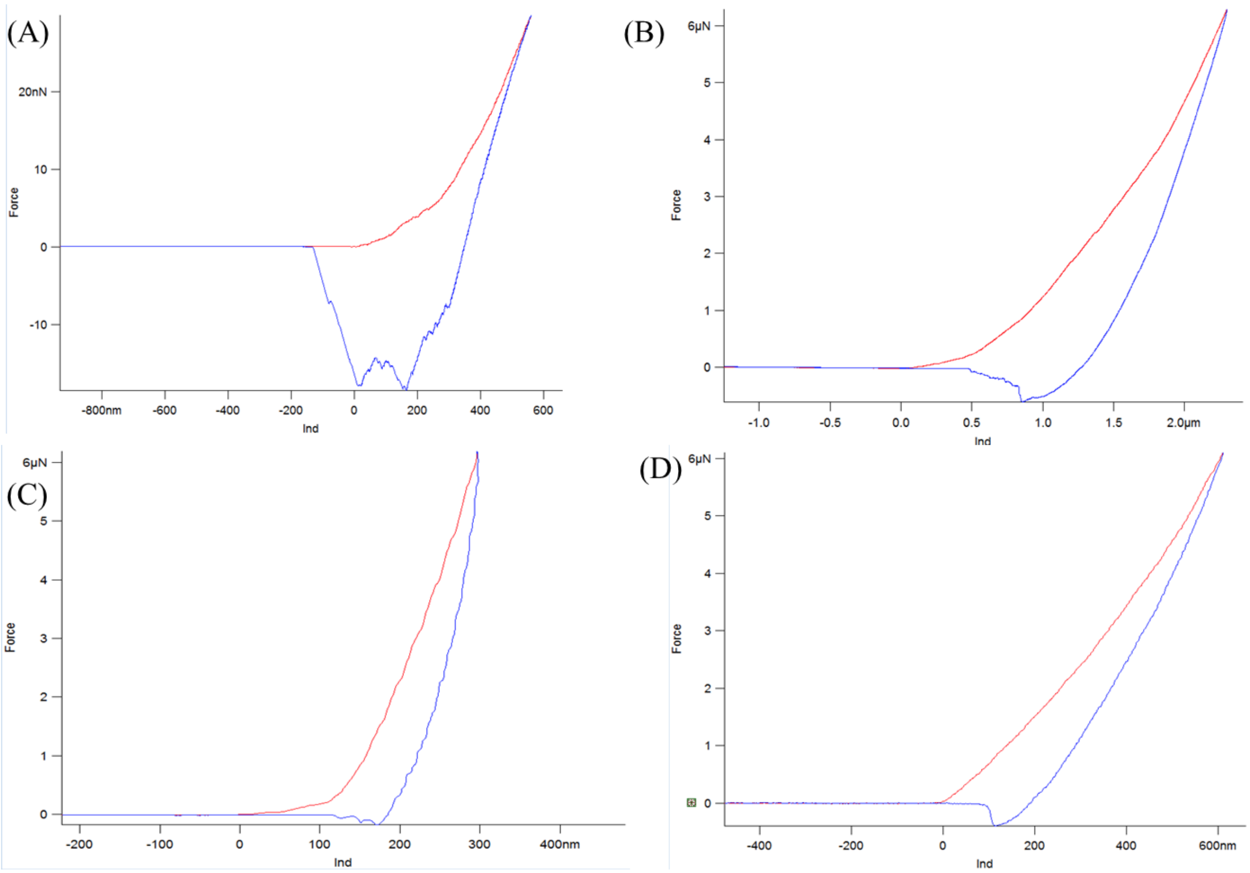

Figure S6 Representative curve of a force versus indentation (Ind) experiment when using (A) a spherical indentor of radius 5.35 micrometer on a bare aerogel, (B) a conical indentor on a bare aerogel, (C) a conical indentor on an aerogel with Ag formed at 0.5 M $AgNO_3$ after exposition of the aerogel to MPTMS, and (D) conical indentor on an aerogel with Ag formed at 0.5 M $AgNO_3$.

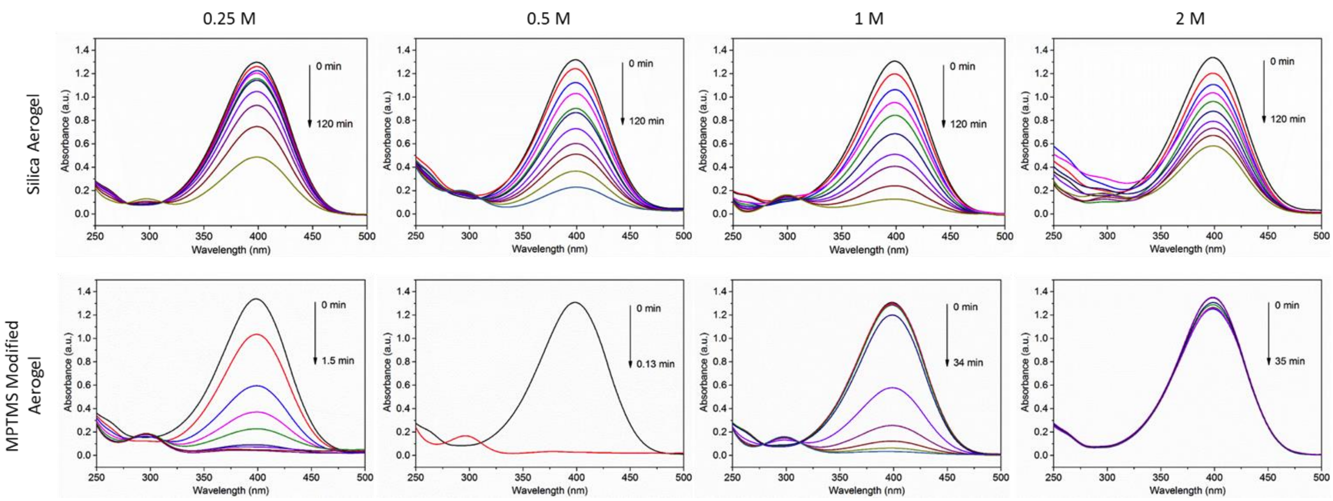

Figure S7 UV-visible spectrophotometer data for the catalysis of the 4-NP as a function of time.

Table S1 Calculated crystallite size of samples with different concentrations of Ag ions

Samples		Crystallite size at different Miller index (hkl) (nm)			
		(111)	(200)	(220)	(311)
Silica	0.25 M	45.89	31.42	38.20	37.90
	0.5 M	41.54	26.26	34.12	34.88
	1 M	52.46	36.86	43.57	42.06
	2 M	50.56	39.96	44.00	44.87
MPTMS modified	0.25 M	23.76	12.13	19.85	22.80
	0.5 M	32.46	20.53	31.54	35.29
	1 M	34.91	22.26	33.75	36.04
	2 M	44.53	35.04	40.49	42.53

Video Supporting Information

The colour of 4-NP aqueous solution with $NaBH_4$ turned from green to no colour. This video indicates the fast reduction of 4-NP with silver-silica aerogels within several seconds upon contacting.
